# Early perinatal outcomes in adolescent pregnancy: a Romanian decade of demographic and social change (2011–2012 vs. 2022–2023)

**DOI:** 10.3389/frph.2026.1808118

**Published:** 2026-04-09

**Authors:** Laura Mihaela Suciu, Vlăduț Săsăran, Irina Bianca Kosovski, Diana Maria Chiorean, Mihai Muntean, Amalia Făgărășan, Claudiu Mărginean, Irina Prelipcean

**Affiliations:** 1 Department of Neonatology, University of Medicine Pharmacy Science and Technology George Emil Palade of Târgu Mureș, Târgu Mureș, Romania; 2 Department of Obstetrics and Gynecology, University of Medicine Pharmacy Science and Technology George Emil Palade of Târgu Mureș, Târgu Mureș, Romania; 3 Department of Pathophysiology, University of Medicine Pharmacy Science and Technology George Emil Palade of Târgu Mureș, Târgu Mureș, Romania; 4 Department of Pediatrics, University of Medicine Pharmacy Science and Technology George Emil Palade of Târgu Mureș, Târgu Mureș, Romania; 5 Department of Neonatology, University of Rochester Medical Center, Golisano Children’s Hospital, Rochester, NY, United States

**Keywords:** adolescent pregnancy, birth rate, neonatal outcomes, repeated cross-sectional study, temporal trends

## Abstract

**Background:**

Pregnancy in adolescence disrupts education, perpetuates economic dependence, and contributes to repeated cycles of poverty. Romania continues to report high adolescent birth rates within the European Union. We evaluated changes in adolescent socioeconomic profiles, perinatal care patterns, and early neonatal outcomes in a Romanian public academic maternity hospital between two snapshots taken ten years apart.

**Materials and methods:**

We conducted a retrospective repeated cross-sectional study of 2237 mother-newborn dyads from two periods: 2011–2012 and 2022–2023. Adolescents (<20 years) were compared with adults aged 25–29 years. The outcomes were delivery mode, preterm birth, low birth weight, small for gestational age (SGA), 5-Apgar score <7, Neonatal Intensive Care Unit (NICU) admission, and breastfeeding initiation. Multivariable logistic models included age group, time period, and their interaction, adjusting for education, antenatal care, and parity

**Results:**

In 2022–2023, adolescent pregnancy was associated with decreased odds of cesarean delivery (OR 0.34, 95% CI 0.26–0.46), SGA (OR 1.76, 95% CI 1.1–2.93), and NICU admission (OR 2.31, 95%CI 1.56–3.01). Breastfeeding initiation declined over time and was less likely among adolescents in the newer period (OR 0.49, 95% CI 0.29–0.84).

**Conclusions:**

Despite improved antenatal care, education and reduced early marriage/cohabiting, adolescents continued to experience higher-risk neonatal outcomes over the past decade. Implementing interventions that strengthen social determinants of health and expand postpartum support is essential.

## Introduction

Adolescent pregnancy, defined as childbirth occurring in mothers before the age of 19 years, remains a major global public health concern and reflects both biological vulnerability and social disadvantage. Early pregnancy is one of the leading global causes of death among girls aged 15–19 years (1), often compounded by inadequate antenatal care, especially in low-resource settings (2)^.^ Physiological immaturity further increases obstetric risks, including higher rates of instrumental delivery (3)^.^ Their infants are at increased risk for preterm birth, intrauterine growth restriction (IUGR), low birth weight (LBW), and other perinatal morbidity (4–7). However, the magnitude of these risks is not uniform, it varies by socioeconomic context, education completion, rural residence, and access to antenatal care. The biological risks are increasingly understood as clinical consequences of social exclusion. Education serves as the primary barrier to early entry into childbearing, and its interruption ensures the intergenerational transmission of poverty. Rural residence and geographic isolation create a “double burden,” where adolescents are more likely to become pregnant and less likely to receive the care necessary to survive the experience (8–12).

Globally, approximately one in ten births occur in adolescents under 19 years, with over 90% in low- and middle-income countries (13). According to the World Health Organization (WHO) latest data, Romania has persistently high adolescent birth rates compared to other European Union (EU) countries (14). While rates declined from 37.22 in 2011 to 33.75 in 2023, concerning trends persist among the youngest group (10–14 years), whose pregnancy rate is 8.5 times higher than the EU average (14). Prior regional studies showed higher prevalence in rural populations with worse neonatal outcomes (15). The specific aims of this study are: 1) to evaluate the differences between early perinatal outcomes among adolescent pregnancies compared to a non-risk adult pregnancy, thereby contextualizing these findings within evolving demographic and social patterns and 2) to quantify temporal changes in perinatal interventions and outcomes over the past decade. We hypothesized that indicators of social vulnerability would improve in the newer cohort, but that adverse neonatal outcomes would persist among adolescent pregnancies compared with adults. Despite abundant literature on adolescent pregnancy outcomes, few studies from Romania have evaluated decade-scale changes using consistent definitions within the same maternity setting, and fewer still have examined whether shifts in social profiles correspond to changes in neonatal outcomes over time. Our repeated cross-sectional design addresses this gap by comparing two periods a decade apart in the same institution. This represents an essential step in identifying whether public health strategies are successfully reaching the most marginalized populations or if the cycle of poverty and poor health continues to stagnate in academic maternity settings.

## Materials and methods

### Study design and setting

This study was conducted at County Hospital Mureș, Târgu Mureș, Romania, a public academically affiliated maternity hospital with approximately 1,600 births annually. Their Level 2 Neonatal Intensive Care Unit (NICU) provides all categories of special care for babies born ≥ 32 weeks but transfers babies requiring complex or longer-term intensive care to a regional tertiary NICU hospital. Because each period represents a distinct set of pregnancies drawn from the same underlying hospital population, rather than longitudinal follow-up of the same individuals, we used a retrospective repeated cross-sectional design. The study follows STROBE guidelines (16).

### Participants and cohorts

**Figure 1:**
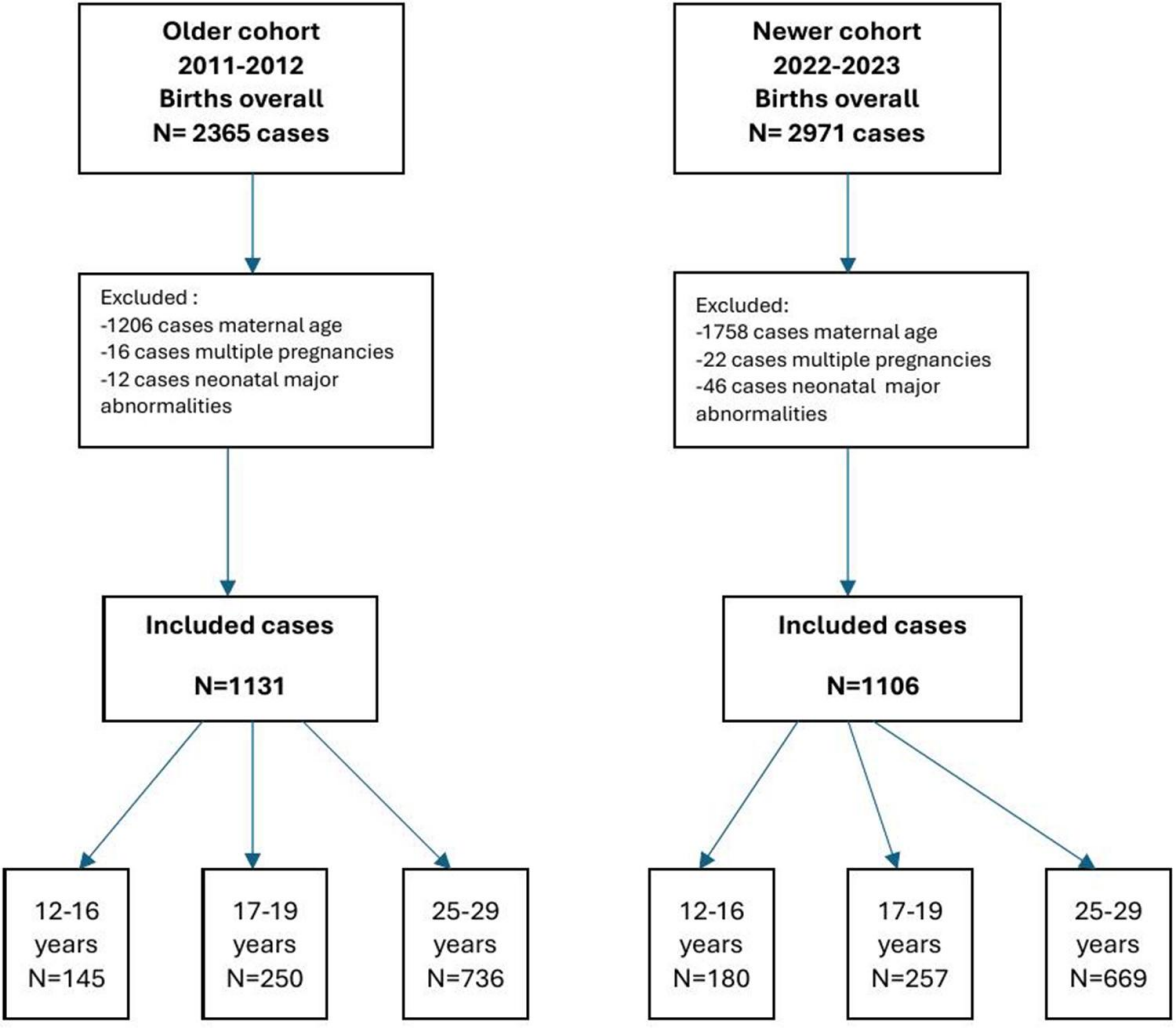
Flow chart of study participants.

A total of 2,237 mother-newborn dyads were identified from January 2011- December 2012 (older cohort) and January 2022 - December 2023 (newer cohort). Eligible participants included all adolescent mothers (12–19 years) and a comparison group of adults (25–29 years) representing the national peak fertility age range according to the Romanian National Institute of Statistics (17). Furthermore, the adolescent mothers were divided into two groups: younger adolescents (up to 16 years) and older adolescents (up to 19 years). A detailed overview of recruitment, exclusions, and analytic samples is shown in Figure 1.

### Exclusion criteria

To establish a low-risk pregnancy at peak fertility age cohort we used the following exclusion criteria: identified major pre-existing illness based on the information recorded in medical files (i.e., malignancies, autoimmune diseases, neurological diseases, ulcerative colitis, asthma, hematologic diseases, and hypothyroidism), multiple gestation, incomplete documentation, and missing first-trimester ultrasound dating.

Infant exclusions: <32 weeks’ gestation, major anomalies, outborn status, and those who needed transfers to a regional tertiary NICU hospital.

### Variables and definitions

Maternal demographic variables included age, rural residence, education, marital/cohabitation status, and self- reported substance use. Obstetric variables included parity, antenatal visits, delivery mode, breech presentation, meconium-stained amniotic fluid, and nuchal cord. Fetal distress was defined as nonreassuring fetal heart rate category II or III according to ACOG criteria (18). Neonatal variables included gestational age, birth weight, Apgar scores, SGA)/LGA status, mode of delivery, NICU admission, and breastfeeding initiation. Maternal coexistent conditions such as anemia, gestational hypertension/preeclampsia, diabetes and postpartum hemorrhagic were not consistently documented in the 2011–2012 dataset; therefore, these variables could not be harmonized across both cohorts and were not included in adjusted models.

Data were extracted from hospital records using consistent definitions across cohorts (definitions and analytical framework were outlined in a prior report) (19).

### Ethics

The study was conducted in accordance with the Declaration of Helsinki. Ethical approval was granted by the Ethics of Research of University of Medicine, Pharmacy Science, and Technology George Emil Palade of Targu Mures, Târgu Mureș, Romania (IRB 2205/March 15th, 2023).

### Sample size and power/precision

Because this was a retrospective study using all eligible deliveries within the defined periods, no *a priori* sample size calculation was performed. We report effect sizes with 95% confidence intervals to emphasize precision.

### Statistical analysis

Descriptive analyses examined sociodemographic factors among young, older adolescents and adults across the two time periods, yielding six independent groups. The outcomes were described as categorical variables and were compared using chi-square or Fisher's exact tests, as appropriate. Significant results were explored through *post-hoc* standardized residual analysis to detect which groups contributed to the overall association. Statistical significance was set at *α*= 0.05 (two-tailed). We conducted multivariable logistic regression using a generalized linear model (binomial distribution; logit link) to calculate odds ratio (ORs) and 95% CI for binary outcomes. The perinatal outcome (cesarean delivery, preterm birth) and neonatal outcome (SGA, LBW, low Apgar score, NICU admission and successful breastfeeding) being the dependent variable and key sociodemographic and health service-related confounders (education, antenatal care and parity). Covariates were selected given their observed variability and theoretical relevance in reproductive health. The predictors included age group, time period, and age-by-time interaction (interpreted as differential change over time) added to evaluate whether temporal changes differed across age categories. In the generalized linear model, Age specific effect ORs were calculated using the adult groups in the corresponding time cohort as the reference. Time effect ORs showed if time itself is associated with a change in the outcomes and the reference was earlier time 2011–2012. The third predictor, Age-by-Time interaction was used to estimate differential temporal slopes between age groups. Specifically, a significant interaction OR (positive or negative) indicates that the association between time and the outcome differs by age group, whereas a non-significant interaction implies comparable slopes of the trend across groups and the reference were adults admitted in the older cohort. Type III Wald chi-square tests evaluated predictor significance. A two-sided *p*-value ≤0.05 was considered statistically significant. Analyses were performed in SPSS (v21.0).

## Results

There were 2,237 mother-newborn dyads in the data set, of which 1,131(50.6%) in the oldest cohort and 1,106 (49.4%) in the newest cohort. Among adolescent mothers, 145(36.7%) included in the older cohort and 257 (41.2%) included in the newest cohort were younger than 17 years. The mean adolescents maternal age was 16.9 (SD = 1.5) years in the older cohort and 16.8 (SD = 1.7) years in the newer cohort. The mean adults maternal age was 26.8 (SD = 1.4) years in the older cohort and 27.3 (SD = 1.4) years in the newer cohort. Overall, 832(37.2%) women were mothers aged 12–19 years and 1,405(62.8%) mothers aged 25–29 years.

### Demographic characteristics

Adolescent mothers had fewer years of education in both cohorts (*p* < 0.05), with improvement over time in age-appropriate education, yet persistent differences compared with adult counterparts. Fewer adolescents were married or cohabiting in 2022–2023. Self-reported alcohol/drug misuse increased in the newer cohort, particularly among younger adolescents. Antenatal visit frequency improved across all groups, though remained lower among adolescents (Table 1).

**Table 1:** Differences in baseline characteristics among groups with different maternal age and admission time.

Variables	Older cohort			Newer cohort			
	12–16 years *N* = 145	17–19 years *N* = 250	Adults *N* = 736	12–16 years *N* = 180	17–19 years *N* = 257	Adults *N* = 669	*p*
Mean maternal age	15.2 (0.8)	17.9 (0.8)	26.8^a^ (1.4)	15.1 (0.9)	18.1 (0.8)	27.3^a^ (1.4)	.001
Rural residence	110 (75.8)^d^	170 (68)	397 (53.9)^c^	104 (57.7)	184 (71.6)^d^	391 (58.4)	.001
Maternal education, years	4.9 (2.9)	5.8 (3.4)	9.8 (4.1)	4.1^a^^,^^b^ (2.8)	5.5 (3.7)	12.1^a^ (4.5)	.001
Age-appropriate education	48 (33.1)^c^	28 (11.2)^c^	428 (58.1)	32 (17.8)^c^	86 (33.5)^c^	555 (82.9)^d^	.001
Child parents Age- gap, years	5.5^a^^,^^b^ (3)	4.6^a^^,^^b^ (3.7)	4^b^ (3.8)	1.7 (3.1)	1.2 (2.9)	0.9 (2.5)	.001
Married/cohabiting	109 (75.1)^d^	121 (48.4)	491 (66.7)	143 (79.4)^d^	113 (43.9)^c^	520 (77.7)	.001
Abortion history	6 (4.1)^c^	38 (15.2)	254 (34.5)	18 (10)^c^	57 (22.2)	192 (28.7)^d^	.001
Parity 1 2 3+	126 (86.9) 18 (12.4) 1 (0.7)	150 (60) 77 (30.8) 23 (9.2)	342 (46.3) 224 (30.4) 170 (23.1)^d^	125 (69.4) 39 (21.7) 0	146 (56.8) 100 (38.9)^d^ 26 (10.1)	271 (40.5)^c^ 139 (31.8) 26 (3.9)	.001
Smoking	19 (13.1)^c^	69 (27.6)	188 (25.5)	40 (22.2)	97 (37.7)^d^	172 (25.7)	.001
Alcohol/drug	10 (6.8)	16 (6.4)	42 (5.7)	28 (15.5)^d^	32 (12.5)^d^	58 (8.7)	.001
Antenatal care	59 (40.7)^c^	130 (52)	398 (54.1)	89 (49.4)^c^	225 (87.5)	611 (91.3)^d^	.001
Gestational age, weeks	38.2 (1.3)	38.1 (1.4)	38.4 (1.3)^a^	38.5 (1.3)	38.5 (1.5)	38.7 (1.4)^a^	.001
Birth weight, grams	3,038 (579)	3,057 (568)	3,308^a^ (531)	3,000 (444)	3,039 (486)	3,296^a^ (454)	.001

Values are presented as mean (SD) and frequency (percentages).

The values are results of chi square test of independence or Fisher exact test as appropriate for categorical variables and linear regression for continuous variables among six independent groups.

Cells without superscript shows no statistically significant deviation.

^a^ significant across adolescents and adults.

^b^ significant across older and newer cohort.

^c^ observed frequency statistically significantly lower than expected *α* = 0.05 (two-tailed).

^d^ observed frequency statistically significantly higher than expected *α* = 0.05 (two-tailed).

### Obstetrical outcomes

Adolescents had fewer induced abortions then adults, however elective termination increased overtime among adolescents (*p* < 0.05). Breech presentation, nuchal cord, preterm birth, and vaginal delivery declined over time in both age groups (*p* < 0.05).

### Neonatal outcomes

Newborns of adolescent mothers had lower birth weight and higher rates of LBW and SGA compared with adults. LGA and birth trauma were more prevalent among adult mothers form the newer cohort compared to adults included in the older cohort. Low Apgar scores (< 7 at 5 min) were more common in infants of adolescent mothers but improved across time. NICU admission rates increased in the newer cohort for both age groups. Breastfeeding initiation declined over time in both adolescents and adults (Table 2).

**Table 2:** Difference in perinatal outcomes among groups with different maternal age and admission time.

	Older cohort			Newer cohort				
Variables	12–16 years	17–19 years	Adults	12–16 years	17–19 years	Adults	*Χ* ^2^	*p*
Cesarean section	16^a^ (11)	35^a^ (14)	152 (20.6)	31^a^ (17.2)	58 (22.6)	283^b^ (42.3)	129.8	.001
Breech fetal	5 (3.4)	16 (6.4)	53 (7.2)^b^	9 (5)	8 (3.1)	18 (2.7)^a^	12.91	.024
Fetal distress	3 (2.1)	6 (2.4)	37^b^ (5)	9^b^ (5)	8^b^ (3.1)	7^a^ (1)	121.1	.001
Nuchal cord	33 (22.7)	62 (24.8)	195 (26.5)^b^	33 (18.3)	41 (15.9)^a^	74 (11)	20.87	.001
Meconium aspiration	0	2 (0.8)	12 (1.6)	4 (2.2)	5 (1.9)	5 (0.7)	9.4	.09
Preterm delivery	16 (11)	30^b^ (12)	63 (8.6)	14 (7.8)	17 (6.6)	39^a^ (5.8)	12.5	.03
LBW	17 (11.7)	42 (16.8)^b^	67 (9.1)	22 (12.2)^b^	32 (12.5)	29 (4.3)^a^	38.6	.001
SGA	26 (17.9)	21 (8.4)	79 (10.7)^a^	39 (21.7)^b^	63 (24.5)^b^	60 (9)^a^	65.5	.001
LGA	4 (2.7)	10 (4)	46 (6.2)	4 (2.2)^a^	7 (2.7)^a^	58 (8.7)^b^	23.1	.001
APGAR < 7	4 (2.8)	8 (3.2)^b^	8 (1.1)	0 (0)	8 (3.1)^b^	7 (1)	14.8	.008
Birth trauma	6 (4.1)	7 (2.8)	31 (4.2)	21 (11.7)	24 (9.3)	82 (12.3)^b^	11.2	.046
NICU admission	10 (6.9)	14 (5.6)	37^a^ (5)	27^b^ (15)	46^b^ (17.9)	47 (7)	104.5	.001
Breast feeding	137^b^ (94.5)	226^b^ (90.4)	605 (82.2)	124 (68.9)	192 (74.7)	440^a^ (65.9)	144.8	.001

Data are presented as frequency (percentages).

Values are the results of chi-square test continuous variables.

Cells without superscript shows no statistically significant deviation.

LBW, low birth weight; SGA, small for gestational age; LGA, large for gestational age; NICU, neonatal intensive care unit.

^a^ observed frequency statistically significantly lower than expected *α* = 0.05 (two-tailed).

^b^ observed frequency statistically significantly higher than expected *α* = 0.05 (two-tailed).

After adjustment, adolescents had lower odds of cesarean delivery in the newer period, with a stronger upward temporal pattern interaction (OR 1.55, 95% CI 1.08–2.39; *p* = 0.04). Adolescent pregnancy remained associated with SGA (OR 1.76, 95% CI 1.1- 2.93; *p* = 0.02) and NICU admission (OR 2.31, 95% CI 1.56–3.01, *p* = 0.02). There was no adjusted differences in temporal risk for preterm birth, low birth weight, or low Apgar score in the newer cohort. Adolescents had lower odds of breastfeeding initiation in the newer period with differential temporal patterns compared with adults (OR 0.49, 95% CI 0.29–0.84; *p* = 0.01) (Table 3).

**Table 3:** Adjusted odds ratio for perinatal outcomes among adolescent mothers .

Variable	Older cohort^a^ Age effect OR[95%CI], p	Newer cohort^a^ Age effect OR[95%CI], p	Time effect^b^ OR[95%CI], p	Differential change overtime^c^ OR[95%CI], p
Cesarean	0.53 [0.41–0.83] 0.001	0.34 [0.26–0.46] 0.001	1.54 [1.07–2.23] 0.019	1.55 [1.08–2.39] 0.04
Preterm	1.47 [0.94–2.73] 0.41	0.71 [0.47–1.06] 0.09	0.57 [0.35–0.93] 0.57	1.14 [0.61–2.14] 0.68
LBW	1.75 [1.20–2.54] 0.002	0.54 [0.35–0.82] 0.004	1.04 [0.68–1.58] 0.84	0.59 [0.31–1.11] 0.11
SGA	1.82 [1.11–2.94] 0.04	1.32 [1.22–2.45] 0.001	1.98 [1.68–2.41] 0.009	1.76 [1.1–2.93] 0.02
Low APGAR	2.85 [1.13–7.03] 0.006	0.56 [0.20–1.57] 0.27	0.96 [0.34–2.66] 0.94	0.61 [0.15–2.41] 0.49
NICU	1.21 [0.72–2.07] 0.2	2.37 [1.25–3.55] 0.001	3.93 [2.12–7.15] 0.001	2.31 [1.56–3.01] 0.03
Breastfeeding	2.45 [1.63–3.69] 0.003	1.47 [1.06–1.88] 0.001	0.35 [0.27–0.45] 0.001	0.49 [0.29–0.84] 0.01

OR, Odds ratio; CI, confidence interval; LBW, low birth weight; NICU, neonatal intensive care unit; SGA, small for gestational age.

^a^ Reference group=adults group admitted in the corresponding cohort.

^b^ Reference group=earlier time group.

^c^ Reference group=adults admitted in the earlier time group.

## Discussion

This study provides a single-center Romanian comparison of adolescent pregnancy spanning two intervals a decade apart, highlighting shifts in demographic characteristics alongside persistently adverse neonatal outcomes. Consistent with prior findings, adolescent mothers were more likely to live in rural areas, have lower education completion, have older partners, and be single at the time of delivery compared with adults. While educational achievement and antenatal care improved, markers of social vulnerability persisted, and self-reported substance use increased. The decline in early marriage/cohabitation likely reflects broader socio-cultural change; however, it may also increase economic weakness when not paired with adequate social support. In our study, induced abortion rates declined among adults but increased among adolescents in the newer cohort, while parity was paradoxically higher among the newer adolescents than earlier adolescents. This pattern raises the possibility that repeated adolescent pregnancy may be linked to social identity and status, a phenomenon described in other settings (20, 21). Because the present study was not designed to measure these mechanisms directly, this interpretation should be framed as a hypothesis-generating explanation rather than a conclusion.

After adjustment, adolescent pregnancy was associated with decreased odds of cesarean delivery compared with adults, and adolescents exhibited a stronger upward time trend. This pattern mirrors global patterns and may reflect evolving obstetric practice rather than maternal risk alone (22–25).

While global meta-analyses and regional studies in Romania (26–28) consistently identify adolescent pregnancy as a significant predictor of preterm birth, with rates reaching 30.5% in southeastern and 18% in central regions cohorts, this study found no independent association after adjustment. This divergence likely reflects specific design and referral differences, as the exclusion of births before 32 weeks preferentially removes the very preterm deliveries that typically drive risk estimates in broader datasets (9, 29, 30). The finding that adolescent pregnancy remains associated with impaired fetal growth (SGA) aligns closely with the wider literature and Romanian data where low education levels and unmonitored pregnancies are primary drivers of reduced birth weight. The persistence of SGA in adjusted models, even as social indicators improved over time, suggests that residual pathways related to nutrition, chronic stress, and constrained resources continue to suppress fetal growth. Although low 5-minute Apgar scores were initially more frequent among adolescents, the improvement observed over time likely reflects better antenatal care engagement, intrapartum management, and educational attainment, all independent predictors of better neonatal outcomes (31). This trend contrasts with the high prevalence of low Apgar scores (32.4%) reported in other very young Romanian cohorts, suggesting that while biological risks persist, they are highly modifiable through targeted care-pathway improvements. Although umbilical cord blood gas analysis beside Apgar score would have been a more precise measure (32), these data were not available for the older cohort, therefore a limitation of our study. NICU admission risk remained higher among adolescents in the newer cohort, suggesting persistent neonatal vulnerability despite improved antenatal engagement (33, 34).

Breastfeeding initiation was high among adolescents in the older cohort but decreased substantially in the newer cohort. After adjustment, adolescent mothers in 2022–2023 were less likely to initiate breastfeeding and showed a stronger negative temporal pattern compared to adults. This mirrors consistent findings of reduced social support and heightened stigma; areas requiring targeted intervention (35–37).

Limitations: Single-center design limits generalizability but supports methodological consistency across cohorts. Exclusion of <32-week deliveries reduce confounding by tertiary referral but may underestimate age-associated risk in extreme prematurity. Because extremely preterm births were excluded, our findings may not be fully representative of the target population of adolescent pregnancies. Key maternal medical variables (anemia, hypertensive disorders, diabetes and post-partum hemorrhagic) and neonatal biochemical measures documenting fetal asphyxia were not consistently available for the 2011–2012 cohort and could not be harmonized, limiting adjustment for these potential confounders. Substance use was self-reported and likely under-ascertained.

Conclusion: Over the past decade, adolescent mothers in this Romanian setting demonstrated improved educational achievement and antenatal care attendance and reduced early marriage/cohabitation, yet adverse neonatal outcomes persisted. These findings support strengthened strategies addressing social determinants, reproductive health education, and continuity coordination of postpartum care.

## Data Availability

The original contributions presented in the study are included in the article/supplementary material, further inquiries can be directed to the corresponding author.
